# Comparison of ultrasound findings of papillary thyroid carcinoma subtypes based on the 2022 WHO classification of thyroid neoplasms

**DOI:** 10.3389/fendo.2024.1434787

**Published:** 2024-08-14

**Authors:** İlhan Hekimsoy, Yeşim Ertan, Gürdeniz Serin, Ahmet Kasım Karabulut, Süha Süreyya Özbek

**Affiliations:** ^1^ Department of Radiology, İzmir Torbalı State Hospital, Izmir, Türkiye; ^2^ Department of Pathology, Ege University Faculty of Medicine, İzmir, Türkiye; ^3^ Department of Radiology, Ege University Faculty of Medicine, Bornova, Türkiye

**Keywords:** WHO classification of thyroid neoplasms, thyroid cancer, papillary, subtype, ultrasonography

## Abstract

**Purpose:**

The present study aimed to analyze and compare sonographic features of papillary thyroid carcinoma (PTC) subtypes to determine whether ultrasound (US) may aid in differentiating particular subtypes.

**Methods:**

This retrospective study enrolled 133 patients diagnosed with 142 histopathologically proven PTCs as per the fifth edition of the World Health Organization classification of thyroid neoplasms between January 2013 and May 2023. US features based on the American College of Radiology and European Thyroid Imaging and Reporting Data Systems (TIRADS), and histopathological characteristics of nodules were assessed and compared.

**Results:**

Histopathological analysis yielded 55 (38.7%) classic PTC, 32 (22.5%) invasive encapsulated follicular variant (IEFV) PTC, 20 (14.1%) oncocytic subtype, 14 (9.9%) non-invasive follicular thyroid neoplasm with papillary-like nuclear features (NIFTP), 11 (7.8%) infiltrative follicular subtype, 7 (4.9%) tall cell subtype, 2 (1.4%) solid subtype, and 1 (0.7%) diffuse sclerosing subtype. The US findings indicating malignancy, such as taller-than-wide shape, irregular margins, echogenic foci, and higher TIRADS categories, were more frequently demonstrated in nodules with classic PTC and the tall cell subtype, in line with their histopathological features. Conversely, IEFV-PTC and NIFTP rarely exhibited these high-risk sonographic features. US appearance of the oncocytic subtype more frequently overlapped with IEFV-PTC, yet hypo/very hypoechoic nodules with larger nodular diameters and higher TIRADS scores may favor the diagnosis of this subtype.

**Conclusion:**

US features of certain subtypes may guide the differential diagnosis regarding shape, margin, echogenic foci, and TIRADS category of nodules; however, definitive subtyping is not yet possible using US images alone.

## Introduction

The most common follicular cell-derived neoplasm is papillary thyroid carcinoma (PTC), accounting for 90% of thyroid malignancies, with an indolent clinical behavior resulting in the highest survival rate (1, 2). Although classic PTC is acknowledged as the archetype, considering PTC subtypes, several other subtypes have been characterized based on their growth patterns, cell types, and stromal changes (3–5). As the histopathological features and molecular profile differ among PTC subtypes, so the clinical course and prognosis will change. Several studies have documented more aggressive behavior and unfavorable prognosis in the tall cell, columnar cell, and hobnail subtypes of PTC (6, 7). Thus, any diagnostic feature demonstrated to increase the likelihood of these aggressive subtypes may be critical in deciding on the clinical management of thyroid nodules. Finding a distinctive imaging characteristic for these subtypes is worthwhile in the era of several well-known guidelines recommending follow-up on subcentimetric yet highly suspicious nodules without performing a biopsy (8–11).

Ultrasonography (US) has a pivotal role in the delineation and characterization of thyroid nodules. The detection of PTC, especially those measuring less than 1 cm, has been mounting in the last decade due to the regular employment of high-resolution US for assessing thyroid abnormalities (12–14). Given the different clinical behaviors and prognosis of PTC subtypes, the 2022 World Health Organization (WHO) classification recommends comprehensive histological subtyping in all PTCs, regardless of size, to individualize the risk stratification in PTC patients (3, 4). Therefore, recognizing the US features of PTC subtypes has become more important in current diagnostic practices.

However, knowledge concerning the differences in US characteristics of PTC subtypes is limited despite US being widely used in the assessment of thyroid nodules. A number of studies have investigated US features of several subtypes, and some have compared these findings with classic PTC (15–21). Nevertheless, based on histopathological analysis, only one study has evaluated the US characteristics and differences between various subtypes (22). To the best of our knowledge, no article has compared US findings between histopathologically proven PTC subtypes based on the 2022 WHO classification of thyroid neoplasms. Thus, the objective of the present paper was to analyze and compare US features of PTC subtypes in light of the novel classification to determine whether US can provide any benefit to the differentiation of particular subtypes.

## Materials and methods

Institutional Review Board approval was obtained for this retrospective study (official decision number: 22-8.1T/19), and the need for written informed consent was waived for the review of medical records, considering the study’s retrospective design.

### Study population

The list of patients who underwent thyroidectomy from January 2013 to May 2023 and were histopathologically proven to have PTC in the pathology department of a tertiary university hospital was reviewed in detail to identify those who had undergone preoperative thyroid US with detailed examination reports and US images obtained in the radiology department of the same institution. In order to ensure that the histopathological diagnosis belongs to a specific nodule, especially in the case of multinodular thyroid glands, the histopathological report of each nodule was compared with preoperative US findings and images, using distinctive features of nodules, including laterality, location, dimensions, and presence of calcification. In the case of any uncertainty regarding the nodule-by-nodule matching process, that nodule was excluded from the study cohort to ensure the radiological-histopathological correlation. For example, in the case of a nodule with malignant histopathological diagnosis in one lobe containing multiple nodules with similar dimensions and US features, the nodule and its lobe were excluded from the study to avoid any doubt about matching. Additionally, nodules lacking subtype analysis or those with inadequate US image quality were not included in the study population. Finally, one patient suffering from the diffuse sclerosing subtype of PTC was excluded from the study group because of the diffuse involvement of one thyroid lobe and isthmus without apparent nodular formation, both on US and histopathological examination.

First, 121 patients harboring 128 nodules with histopathologically proven diagnosis and detailed preoperative US features were enrolled in the study group. Seven of these patients had two different PTC nodules simultaneously, while two of the remaining patients had a nodule with the histopathological diagnosis of “non-invasive follicular thyroid neoplasm with papillary-like nuclear features” (NIFTP) in addition to their nodules with PTC histopathology. Given that NIFTP lesions often sonographically mimic some PTC tumors, especially those with a subsequent histopathological diagnosis of “invasive encapsulated follicular variant papillary thyroid carcinoma” (IEFV-PTC), we decided to include other NIFTP nodules in the study group to enable a better comparison and differential diagnosis of PTC lesions in terms of US features. Thus, 12 additional patients, each harboring a thyroid nodule with the histopathological diagnosis of NIFTP and US features obtained using the above-described matching process, were also included in the study. Ultimately, 142 thyroid nodules with the histopathologic diagnosis of PTC (128 nodules, 90%) or NIFTP (14 nodules, 10%) in a total of 133 patients constituted the final study group.

Among 128 thyroid nodules presenting with the histopathological features of PTC, 55 (43%) nodules were diagnosed as classic PTC, while the histopathological diagnosis, according to the 2022 WHO classification, was one of the different PTC subtypes in 41 nodules including oncocytic (20 nodules, 16%), tall cell (11 nodules, 8%), infiltrative follicular (7 nodules, 5%), solid (2 nodules, 2%), and diffuse sclerosing (1 nodule, 1%) subtypes. The remaining 32 (25%) nodules with histopathological features of PTC were finally diagnosed as “invasive encapsulated follicular variant PTC” (IEFV-PTC), a separate category now isolated from other PTC subtypes in the 2022 WHO classification.

### US examination and image assessment

A single radiologist (SSO) with more than 30 years of experience in thyroid US performed all preoperative thyroid and neck US examinations using one of two US devices (the Acuson S1000™ or S2000™, Siemens Medical Solutions USA, Inc, Mountain View, CA, USA) equipped with 4- to 9-MHz, 5- to 15-MHz or 5.5- to 18-MHz linear array transducers. The thyroid gland and all neck compartments were thoroughly investigated during the US examination. After delineation of a thyroid nodule, its location in the thyroid gland, diameters in three anatomical planes (i.e., transverse, longitudinal, and anteroposterior), echogenicity (compared to the thyroid parenchyma or the strap muscles), border regularity (smooth, ill-defined, spiculated, microlobulated), and internal composition (solid, mixed predominantly solid or cystic, cystic, spongiform) were assessed. In addition, the presence of echogenic foci comprising macrocalcification, rim calcification or punctate echogenic foci (either colloidal microcrystals adjacent to cystic components or apparently frank microcalcifications in the solid parts) and extra-thyroidal extension were evaluated. Imaging findings were classified based on the American College of Radiology (ACR) and European Thyroid Association guidelines (9, 10). High-quality grayscale images illustrating significant US findings of each nodule and detailed US reports of the patients were digitally stored.

The same radiologist (SSO) who performed the US examination categorized all nodules according to the ACR- and EU-TIRADS guidelines based on their sonographic features (9, 10).

### Histopathological analysis

An experienced pathologist in thyroid neoplasms reviewed all slides of nodules diagnosed as PTC to determine their subtype based on the 2022 WHO classification (3, 4). Classic PTC was designated when tumor cells with specific nuclear characteristics, including enlargement, elongation, grooving, and chromatin clearing/margination, line well-developed papillae. The tumor was further stratified as encapsulated classic PTC if a thick fibrous capsule surrounding the whole nodule was evident. A tumor with an infiltrative follicular growth pattern, florid nuclear atypia, and lacking prominent papillae formation was diagnosed as the infiltrative follicular subtype of PTC. A tumor was classified as the tall cell subtype of PTC when more than 30% of tumor cells were three times taller than wide and had eosinophilic cytoplasm along with conventional nuclear features of PTC. The solid PTC subtype was characterized by solid, trabecular, or nested growth patterns constituting more than 50% of the tumor. The diffuse sclerosing subtype of PTC was identified with diffuse involvement of thyroid parenchyma, dense fibrous stroma, widespread lymphatic infiltration, and frequent psammoma bodies. A tumor composed of more than 75% oncocytic cells with classic PTC nuclear features and well-formed papillae was defined as the oncocytic PTC subtype. An infiltrative tumor harboring all characteristics of the classic PTC apart from papillae or an invasive encapsulated tumor mimicking follicular thyroid carcinoma except bearing PTC nuclear features was marked as the IEFV-PTC. After meticulous microscopic examination, a tumor without invasive growth and having less than 1% true papillae and PTC nuclear characteristics was reclassified as NIFTP since these nodules had been accepted as the follicular variant of PTC before 2016 (23).

The pathology reports of all nodules were evaluated to record the tumor’s largest diameter, localization, multicentricity, presence of a capsule, tumor’s margin regularity, capsular/lymphatic/vascular or perineural invasion, extrathyroidal extension, presence of mitosis (<5 mitoses/2mm^2^) and necrosis, surgical margins, and thyroid parenchymal features.

### Statistical analysis

Categorical variables are presented as counts and percentages, while continuous variables are demonstrated as mean and standard deviation (SD) or median and interquartile range (IQR) based on the normality assumption assessed by the Shapiro-Wilk test. The one-way ANOVA and Kruskal-Wallis tests were conducted to analyze the age and size differences among histopathological subtypes, respectively. The diffuse sclerosing subtype was excluded from the age and size comparison analysis owing to the small sample size (n=1). Pearson’s chi-square test or Fisher’s exact test was used to assess whether any correlation was present between the sonographic findings and histopathological features and to detect the difference in sonographic findings of histopathological subtypes. The cells producing a significant Chi-square result on r x c contingency tables were identified using adjusted residuals. Cramer’s V was calculated for the effective size, indicating the strength of the association, which was categorized as follows: weak (0.1 to 0.29), moderate (0.3 to 0.49), or strong (0.5 or greater) (24). The statistical analysis was conducted using IBM-SPSS 25.0 for the Windows software package (IBM Corp., Armonk, NY, USA). Statistical significance was defined as a P-value of less than 0.05 for all statistical methods.

## Results

Overall, 133 patients, including 29 (21.8%) men with 31 (21.8%) nodules and 104 (78.2%) women with 111 (78.2%) nodules, with a mean age of 45.13 ± 15.11 years, ranging from 13 to 77 years, were enrolled in the study. The demographic characteristics and maximum nodular diameters of the study population are presented in **Table 1**. Although there was no significant difference between the mean ages of patients with different histopathological diagnoses, the highest mean age was detected in the patients with oncocytic subtype (53.34 ± 15.35 years; range, 19 to 77 years) while the lowest age was observed in a 35-year-old patient with diffuse sclerosing subtype, who was excluded from statistical comparison of mean ages since there was only one case. Among the PTC subtypes, NIFTP and IEFV-PTC, the distribution of patients was not statistically significant in terms of gender.

**Table 1:** Demographic characteristics and maximal nodular diameters of the study population.

Variable	Classic PTC	IEFV-PTC	Oncocytic subtype	NIFTP	Tall cell subtype	Infiltrative follicular subtype	Solid subtype	Diffuse sclerosing subtype	P-value
Age (year)	41.9 ± 13.3	46.4 ± 15.1	53.3 ± 15.3	43.2 ± 19.1	50.3 ± 18.8	40.8 ± 6.9	42.4 ± 17.5	35^⁎^	0.139
Sex									0.121
Male (n=29)	7 (13.2)	12 (38.7)	5 (27.8)	2 (15.4)	3 (33.3)	0	0	0	
Female (n=104)	46 (86.8)	19 (61.3)	13 (72.2)	11 (84.6)	6 (66.7)	6 (100)	2 (100)	1 (100)	
Number of nodules									
Male (n=31)	7 (22.6)	13 (41.9)	5 (16.1)	2 (6.5)	4 (12.9)	0	0	0	
Female (n=111)	48 (43.2)	19 (17.1)	15 (13.5)	12 (10.8)	7 (6.3)	7 (6.3)	2 (1.8)	1 (0.9)	
Total (n=142)	55 (38.7)	32 (22.5)	20 (14.1)	14 (9.9)	11 (7.8)	7 (4.9)	2 (1.4)	1 (0.7)	
Maximal nodular diameter (mm)									
US	10.0 (7.0-16.0)	28.5 (20.0-40.0)	22.5 (13.3-26.8)	16.0 (8.8-35.5)	12.0 (8.0-20.0)	14.0 (10.0-15.0)	63.5 (47.0-80.0)^⁎⁎^	14^⁎^	<0.001
Histopathological	10.0 (7.0-15.0)	25.0 (19.3-34.3)	20.0 (13.0-21.8)	17.0 (8.3-26.3)	12.0 (9.0-18.0)	8.0 (4.0-12.0)	46.5 (35.0-58.0)^⁎⁎^	11^⁎^	<0.001

Values are presented as mean ± SD, median (IQR), or count (%).

PTC, papillary thyroid carcinoma; IEFV, invasive encapsulated follicular variant; NIFTP, non-invasive follicular thyroid neoplasm with papillary-like nuclear features; US, ultrasound.

^⁎^The diffuse sclerosing subtype was excluded from the age and size comparison analysis owing to the small sample size.

^⁎⁎^Maximal nodular diameters of the solid subtype are presented as median (range) since there are only two variables.

The maximum nodular diameters measured either using sonographic [15 mm; 9-27 (median; IQR)] or histopathological technique [14 mm; 8-24 (median; IQR)] differed significantly among the PTC subtypes and IEFV-PTC. The mean value of US-measured diameters of classic PTC lesions was significantly smaller than those of IEFV-PTC and the oncocytic subtype lesions (multiple pairwise comparisons; P<0.001 and 0.033, respectively). Similarly, the mean histopathological diameters of the infiltrative follicular subtype were significantly smaller than those of IEFV-PTC and the solid subtype (P=0.002 and 0.040, respectively), while the IEFV-PTC lesions were significantly larger than that of classic PTC lesions (P<0.001).

In the comparison of sonographic features and histopathological findings, a “taller-than-wide shape” on US examination was found to be more frequently associated with infiltrative borders and lack of a capsule in histopathological assessment (P<0.001). Similarly, nodules with lobulated or irregular sonographic margins frequently exhibited infiltrative borders and were less often shown to have a capsule in pathological examination (P<0.001). Histopathologically reported lymphatic invasion was found to be significantly associated with lobulated or irregular margins, a taller-than-wide shape, marked hypoechogenicity, and microcalcifications in US examination, as well as the EU- or ACR-TIRADS score of 5. The presence of microcalcification was also significantly associated with histopathological findings of infiltrative borders, capsule infiltration, and intrathyroidal invasion. Multicentricity, necrosis, vascular invasion, extrathyroidal extension, and perineural invasion did not correlate with any sonographic features.

The US characteristics of nodules in the study population are summarized in **Table 2**. A significant difference in shape was detected in the nodules with histopathologic diagnoses of different PTC subtypes and NIFTP (P<0.001, Cramer’s V=0.427). The “taller-than-wide” shape was more frequently observed in the classic PTC (41.8%) and the tall cell subtype (54.5%), while 90.6% of the IEFV-PTCs and 95.0% of the oncocytic subtypes exhibited a “wider-than-tall” shape. The nodule margin also varied significantly among the PTC subtypes, NIFTP and IEFV-PTC (P<0.001, Cramer’s V=0.388). Lobulated or irregular margins were predominantly seen in classic PTC (78.2%) and the tall cell subtype (81.8%), whereas NIFTP, IEFV-PTC, and the oncocytic subtype demonstrated a greater tendency to have smooth margins (78.6%, 68.8%, and 75%, respectively). Echogenic foci were the other US feature differing significantly among histopathological subtypes (P=0.007, Cramer’s V=0.343) and were detected more frequently in classic PTC (65.5%), while the oncocytic subtype occasionally presented with echogenic foci (20%). Similarly, the highest prevalence of intranodular microcalcification was identified in classic PTC. In contrast, the oncocytic subtype and NIFTP presented without microcalcification, while only one nodule with microcalcification was observed in patients having IEFV-PTC (P<0.001, Cramer’s V=0.547). Both EU- and ACR-TIRADS categories varied considerably among different histopathological subtypes (P<0.001 for both classification systems, Cramer’s V=0.508 and 0.418, respectively). Most of the nodules with a histopathological diagnosis of classic PTC and the tall cell subtype were rated in category 5 using the EU- and ACR-TIRADS. Conversely, the IEFV-PTC was more frequently assigned to EU- and ACR-TIRADS category 3, as were NIFTP nodules. Remarkably, nodules classified as oncocytic subtype were mostly classified under category 4 in both stratification systems.

**Table 2:** Comparison of the sonographic features of the nodules having different histopathologic diagnoses.

Variable	Classic PTC (n=55)	IEFV-PTC (n=32)	Oncocytic subtype (n=20)	NIFTP (n=14)	Tall cell subtype (n=11)	Infiltrative follicular subtype (n=7)	Solid subtype (n=2)	Diffuse sclerosing subtype (n=1)	P-value	Cramer’s V
Composition									0.106	
Solid or almost completely solid	52 (94.5)	28 (87.5)	19 (95.0)	10 (71.4)	10 (90.9)	6 (85.7)	1 (50.0)	1 (100)		
Mixed cystic and solid	3 (5.5)	4 (12.5)	1 (5.0)	4 (28.6)	1 (9.1)	1 (14.3)	1 (50.0)	0		
Echogenicity									0.073	
Hyper or isoechoic	11 (20.0)	15 (46.9)	7 (35.0)	9 (64.3)	1 (9.1)	1 (14.3)	0	0		
Predominantly isoechoic with hypoechoic areas	4 (7.3)	4 (12.5)	2 (10.0)	0	1 (9.1)	0	1 (50.0)	0		
Predominantly hypoechoic with isoechoic areas	2 (3.6)	1 (3.1)	1 (5.0)	0	1 (9.1)	1 (14.3)	0	0		
Hypoechoic	31 (56.4)	12 (37.5)	8 (40.0)	5 (35.7)	6 (54.5)	4 (57.1)	1 (50.0)	1 (100)		
Very hypoechoic	7 (12.7)	0	2 (10.0)	0	2 (18.2)	1 (14.3)	0	0		
Shape									<0.001	0.427
Taller-than-wide	23 (41.8)^⁎^	3 (9.4)^⁎^	1 (5.0)^⁎^	1 (7.1)	6 (54.5)^⁎^	1 (14.3)	0	0		
Wider-than-tall	32 (58.2)^⁎^	29 (90.6)^⁎^	19 (95.0)^⁎^	13 (92.9)	5 (45.5)^⁎^	6 (85.7)	2 (100)	1 (100)		
Margin									<0.001	0.388
Smooth	11 (20.0)^⁎^	22 (68.8)^⁎^	15 (75.0)^⁎^	11 (78.6)^⁎^	2 (18.2)	2 (28.6)	2 (100)	0		
Ill-defined	1 (1.8)	0	0	0	0	0	0	0		
Lobulated or irregular	43 (78.2)^⁎^	10 (31.2)^⁎^	5 (25.0)^⁎^	3 (21.4)^⁎^	9 (81.8)^⁎^	5 (71.4)	0	1 (100)		
Echogenic foci									0.007	0.343
Present	36 (65.5)^⁎^	12 (37.5)	4 (20.0)^⁎^	6 (42.9)	7 (63.6)	3 (42.9)	1 (50.0)	1 (100)		
None or large comet tail artifacts	19 (34.5)^⁎^	20 (62.5)	16 (80.0)^⁎^	8 (57.1)	4 (36.4)	4 (57.1)	1 (50.0)	0		
Macrocalcifications									0.513	
Present	11 (20.0)	4 (12.5)	3 (15.0)	2 (14.3)	4 (36.4)	2 (28.6)	1 (50.0)	0		
Absent	44 (80.0)	28 (87.5)	17 (85.0)	12 (85.7)	7 (63.6)	5 (71.4)	1 (50.0)	1 (100)		
Rim calcifications									0.592	
Present	2 (3.6)	0	0	1 (7.1)	0	0	0	0		
Absent	53 (96.4)	32 (100)	20 (100)	13 (92.9)	11 (100)	7 (100)	2 (100)	1 (100)		
Colloid microcrystals									0.212	
Present	4 (7.3)	7 (21.9)	1 (5.0)	4 (28.6)	2 (18.2)	1 (14.3)	0	0		
Absent	51 (92.7)	25 (78.1)	19 (95.0)	10 (71.4)	9 (81.8)	6 (85.7)	2 (100)	1 (100)		
Microcalcifications									<0.001	0.547
Present	27 (49.1)^⁎^	1 (3.1)^⁎^	0^⁎^	0^⁎^	3 (27.3)	1 (14.3)	0	1 (100)		
Absent	28 (50.9)^⁎^	31 (96.9)^⁎^	20 (100)^⁎^	14 (100)^⁎^	8 (72.7)	6 (85.7)	2 (100)	0		
EU-TIRADS									<0.001	0.508
3	7 (12.7)^⁎^	18 (56.3)^⁎^	5 (25.0)	7 (50.0)^⁎^	1 (9.1)	1 (14.3)	0	0		
4	1 (1.8)^⁎^	8 (25.0)	9 (45.0)^⁎^	3 (21.4)	0	3 (42.9)	2 (100)^⁎^	0		
5	47 (85.5)^⁎^	6 (18.7)^⁎^	6 (30.0)^⁎^	4 (28.6)^⁎^	10 (90.9)^⁎^	3 (42.9)	0	1 (100)		
ACR-TIRADS									<0.001	0.418
2	1 (1.8)	2 (6.2)	0	2 (14.3)^⁎^	1 (9.1)	0	0	0		
3	4 (7.3)^⁎^	15 (46.9)^⁎^	5 (25.0)	4 (28.6)	0	1 (14.3)	0	0		
4	9 (16.4)^⁎^	11 (34.4)	13 (65.0)^⁎^	6 (42.8)	0^⁎^	2 (28.6)	1 (50.0)	0		
5	41 (74.5)^⁎^	4 (12.5)^⁎^	2 (10.0)^⁎^	2 (14.3)^⁎^	10 (90.9)^⁎^	4 (57.1)	1 (50.0)	1 (100)		

Values are presented as count (%).

PTC, papillary thyroid carcinoma; IEFV, invasive encapsulated follicular variant; NIFTP, non-invasive follicular thyroid neoplasm with papillary-like nuclear features; ACR-TIRADS, American College of Radiology Thyroid Imaging Reporting and Data System; EU-TIRADS, European Thyroid Association Thyroid Imaging Reporting and Data System.

^⁎^Denoting cells with an adjusted residual value larger than 1.96 or less than -1.96.

The histopathological characteristics of the study population are demonstrated in **Table 3**. In the comparison of histopathological findings of the subtypes, lymphatic invasion was more frequently observed in classic PTC (50.9%) and the tall cell subtype (81.8%), contrasting with IEFV-PTC and the oncocytic subtype (P<0.001, Cramer’s V= 0.614). No lymphatic invasion was detected in NIFTP and the infiltrative follicular subtype, although the adjusted residual value was not significant for the latter due to the small sample size. All IEFV-PTC nodules were encapsulated, as expected, whereas the infiltrative follicular and tall cell subtypes presented without a capsule. Moreover, the oncocytic subtype displayed a greater predisposition to have a capsule, while classic PTC was less frequently encapsulated [n=14 (70.0%) and n=11 (20.0%), respectively; P<0.001, Cramer’s V=0.709]. All IEFV-PTC and NIFTP exhibited smooth borders, unlike the infiltrative follicular and tall cell subtypes, which all presented with infiltrative margins. In addition, smooth borders were noted in 95% of the oncocytic subtype, whereas infiltrative margins were more frequently seen in classic PTC (81.8%) (P<0.001, Cramer’s V=0.861). More than four-fifths (81.8%) of the tall cell subtype nodules exhibited mitosis, as opposed to IEFV-PTC, of which 21.9% presented with mitosis (P=0.002, Cramer’s V=0.397). Lastly, multicentricity was more frequently detected in the tall cell subtype (63.6%; P=0.005, Cramer’s V=0.386).

**Table 3:** Histopathological characteristics of the nodules with different histopathological diagnoses.

Variable	Classic PTC (n=55)	IEFV-PTC (n=32)	Oncocytic subtype (n=20)	NIFTP (n=14)	Tall cell subtype (n=11)	Infiltrative follicular subtype (n=7)	Solid subtype (n=2)	Diffuse sclerosing subtype (n=1)	P-value	Cramer’s V
Border regularity									<0.001	0.861
Smooth	10 (18.2)^⁎^	32 (100)^⁎^	19 (95.0)^⁎^	14 (100)^⁎^	0^⁎^	0^⁎^	2 (100)	0		
Infiltrative	45 (81.8)^⁎^	0^⁎^	1 (5.0)^⁎^	0^⁎^	11 (100)^⁎^	7 (100)^⁎^	0	1 (100)		
Capsule									<0.001	0.709
Present	11 (20.0)^⁎^	32 (100)^⁎^	14 (70.0)^⁎^	9 (64.3)	1 (9.1)^⁎^	0^⁎^	1 (50.0)	0		
Absent	44 (80.0)^⁎^	0^⁎^	6 (30.0)^⁎^	5 (35.7)	10 (90.9)^⁎^	7 (100)^⁎^	1 (50.0)	1 (100)		
Capsule Invasion									<0.001	0.845
Present	5 (45.5)^⁎^	32 (100)^⁎^	13 (92.9)	0^⁎^	0	-	1 (100)	-		
Absent	6 (54.5)^⁎^	0^⁎^	1 (7.1)	9 (100)^⁎^	1 (100)	-	0	-		
Lymphatic invasion									<0.001	0.614
Present	28 (50.9)^⁎^	1 (3.1)^⁎^	2 (10.0) ^⁎^	0^⁎^	9 (81.8)^⁎^	0	0	1 (100)		
Absent	27 (49.1)^⁎^	31 (96.9)^⁎^	18 (90.0) ^⁎^	14 (100)^⁎^	2 (18.2)^⁎^	7 (100)	2 (100)	0		
Vascular Invasion									0.153	
Present	0	0	1 (5.0)	0	1 (9.1)	0	0	0		
Absent	55 (100)	32 (100)	19 (95.0)	14 (100)	10 (90.9)	7 (100)	2 (100)	1 (100)		
Extrathyroidal invasion									0.234	
Present	1 (1.8)	1 (3.1)	0	0	2 (18.2)	0	0	0		
Absent	54 (98.2)	31 (96.9)	20 (100)	14 (100)	9 (81.8)	7 (100)	2 (100)	1 (100)		
Necrosis									-	
Present	0	0	0	0	0	0	0	0		
Absent	55 (100)	32 (100)	20 (100)	14 (100)	11 (100)	7 (100)	2 (100)	1 (100)		
Mitosis^⁎⁎^									0.002	0.397
Present	19 (34.5)	7 (21.9)^⁎^	10 (50.0)	2 (14.3)	9 (81.8)^⁎^	3 (42.9)	2 (100)	1 (100)		
Absent	36 (65.5)	25 (78.1)^⁎^	10 (50.0)	12 (85.7)	2 (18.2)^⁎^	4 (57.1)	0	0		
Multicentricity									0.005	0.386
Present	9 (16.4)	4 (12.5)	7 (35.0)	2 (14.3)	7 (63.6)^⁎^	3 (42.9)	0	1 (100)		
Absent	46 (83.6)	28 (87.5)	13 (65.0)	12 (85.7)	4 (36.4)^⁎^	4 (57.1)	2 (100)	0		
Perineural invasion									0.72	
Present	0	0	0	0	0	1 (14.3)	0	0		
Absent	55 (100)	32 (100)	20 (100)	14 (100)	11 (100)	6 (85.7)	2 (100)	1 (100)		

Values are presented as count (%).

PTC, papillary thyroid carcinoma; IEFV, invasive encapsulated follicular variant; NIFTP, non-invasive follicular thyroid neoplasm with papillary-like nuclear features.

^⁎^Denoting cells with an adjusted residual value larger than 1.96 or less than -1.96.

^⁎⁎^Presence of mitosis indicates <5 mitoses per 2 mm^2^.

## Discussion

Recognizing PTC subtypes has become pivotal after the implementation of active surveillance into clinical practice in low-risk patients with papillary thyroid microcarcinoma, as high-risk subtypes are not suitable for this kind of observational management (8, 13, 25, 26). The 2022 WHO classification of thyroid neoplasms also recommended detailed subtyping in all nodules, considering that not all PTCs have indolent clinical courses, and some subtypes with aggressive biological behaviors (i.e., tall cell, columnar cell, and hobnail subtypes) may exhibit local or distant metastases and recurrence after definitive treatment (4). Therefore, given the fundamental role of US in thyroid nodule interpretation, any US characteristics aiding in differentiating subtypes with unfavorable outcomes from those with indolent clinical behavior will enhance individualized clinical management in patients with PTC.

The present study found no difference among the US features and TIRADS categories of the infiltrative follicular subtype, NIFTP, and IEFV-PTC. Although hypoechoic or markedly hypoechoic nodules, lobulated/irregular margins, the EU-TIRADS category 4-5, and the ACR-TIRADS category 5 nodules were more frequently detected in the infiltrative follicular subtype, the difference did not reach statistical significance, which was probably related to a relatively small sample size of the infiltrative follicular subtype. Similarly, Baek et al. (22) report no significant difference between the US characteristics of the infiltrative and encapsulated follicular variants, yet the study enrolled only four cases of encapsulated variants. In contrast, a significant difference was reported in all US findings in two studies evaluating the infiltrative follicular variant, NIFTP, and IEFV-PTC based on Korean TIRADS (27, 28). Moreover, Kwon et al. (28) demonstrated grayscale histogram analysis as a viable tool for differentiating the infiltrative follicular variant from the IEFV-PTC and NIFTP; however, histogram parameters of the IEFV-PTC and NIFTP were not shown to differ significantly. Similarly, Rosario et al. (29) did not observe a remarkable difference between the US appearance of the IEFV-PTC and NIFTP, which was compared as per the American Thyroid Association classification. In the present study, the IEFV-PTC and NIFTP also exhibited similar US features, such as a wider-than-tall shape, regular borders, solid composition, iso or hyperechogenicity, and lack of microcalcification, which were significantly different from classic PTC in terms of border regularity and presence of microcalcification. Additionally, a wider-than-tall shape was found to be significantly associated with the IEFV-PTC compared to classic PTC. In line with the US findings, TIRADS scores for the IEFV-PTC and NIFTP significantly differed from classic PTC. In contrast, US characteristics of the infiltrative follicular subtype more frequently overlapped with classic PTC, as reported in previous studies (27, 28). Similar to US characteristics, histopathological findings also indicated more favorable biological behavior in the IEFV-PTC and NIFTP, given smooth borders and less frequent lymphatic invasion and mitosis.

In accordance with previous studies (21, 30), the tall cell subtype exhibited a taller-than-wide shape, irregular borders, solid composition, hypoechoic or markedly hypoechoic appearance, and echogenic foci, which in turn was predominantly classified under the EU- and ACR TIRADS 5 in the present study. As Baek et al. (20) reported, no significant difference was demonstrated between the US appearance of the tall cell subtype and classic PTC in the present study. In contrast, Kim et al. (21) reported a frequent capsular location and a higher likelihood of irregular margins depending on the tumor size for the tall cell subtype compared to classic PTC. Moreover, Zang et al. (31) focused on the pathologic basis of US characteristics of the tall cell subtype and found that distinct isoechoic regions along with peripheral hypoechoic halos were related to unevenly distributed fibrous stroma and peripherally located highly cellular epithelium, which was more frequently detected in the tall cell subtype as opposed to classic PTC. Additionally, a thick hypoechoic halo was suggested as a US feature, indicating the aggressive nature of the tall cell subtype since peripherally located tall cells enhance the probability of tumor infiltration into adjacent tissue (31). In a similar vein, we observed lymphatic invasion, mitosis, multicentricity, and infiltrative tumor margins more frequently in tall cell subtypes, which were the histopathological findings indicating aggressive biological behavior.

The oncocytic subtype constituted the largest group among PTC subtypes in the present study, although it was reported to be an uncommon subtype, accounting for 1-11% of PTCs (15). Similar to the literature data (32), the mean age of patients with the oncocytic subtype was detected to be the highest among all PTC subtypes, yet the difference in mean ages between subtypes was not significant. As previously reported, the oncocytic subtype presented with a larger tumor size than classic PTC in our study (33). The oncocytic subtype commonly exhibited a wider-than-tall shape, regular borders, solid composition, and hypo- or iso/hyperechogenicity without microcalcification, and thus, the majority were categorized under the EU- and ACR-TIRADS category 4. US features of the oncocytic subtype were remarkably different from those of classic PTC and the tall cell subtype in terms of shape and border regularity. Additionally, lack of microcalcification may significantly differentiate the oncocytic subtype from classic PTC. However, these US findings overlapped with those of the IEFV-PTC and NIFTP; nevertheless, the EU- and ACR-TIRADS scores of these subtypes varied significantly. As opposed to our results, Baek et al. (22) did not observe any significant differences between the US characteristics of the PTC subtypes, but only two nodules with oncocytic variant were included in that study. In the present study, the oncocytic subtype displayed a smooth border on histopathological examination similar to its US appearance and was encapsulated with frequent capsule invasion, while lymphatic invasion was less frequently detected compared to classic PTC and the tall cell subtype. Gross et al. (34) also reported a high incidence of capsule invasion in the oncocytic variant, suggesting this might be related to a more aggressive disease course than classic PTC. However, recent studies have demonstrated similar clinical outcomes (32, 35). According to our results, the oncocytic subtype should be considered in the differential diagnosis of nodules with larger maximal diameters, smooth borders, that are hypo- or very hypoechogenic, and without microcalcification.

The solid and diffuse sclerosing subtypes are uncommon and have a less favorable prognosis (16). The US features of the solid subtype overwhelmingly overlapped with those of the oncocytic subtype, IEFV-PTC, and NIFTP in the present study. Giorgadze et al. (36) reported similar US findings, except for the dyshesive single-cell cytomorphologic pattern exhibiting irregular borders, representing heterogeneous cytologic characteristics. In the current study, the diffuse sclerosing subtype displayed irregular margins, solid composition, marked hypoechogenicity, and scattered microcalcifications in both thyroid lobes. The snowstorm appearance, denoting scattered microcalcifications, was described as a unique US characteristic to differentiate the diffuse sclerosing subtype, although its US appearance may mimic thyroiditis in the absence of a nodule formation (20). On histopathological examination, mitosis was detected in both subtypes, while multicentricity, and lymphatic invasion were reported in the diffuse sclerosing subtype, signifying the less favorable behavior.

Several limitations should be taken into account while considering our results. First, the retrospective study design may have culminated in selection bias. However, given the rarity of certain subtypes, prospective analysis was not feasible. Second, enrolling only histopathologically confirmed nodules into the study cohort may have introduced sampling bias and caused a relatively high number of the oncocytic subtype, yet histopathology is accepted as a reference standard to diagnose PTC subtypes. Third, a single radiologist performed all US examinations and interpreted the images, leading to the lack of interoperator reproducibility assessment. In addition, intraoperator reproducibility was not considered in the present study. Fourth, the small sample size excluded specific subtypes (i.e., columnar cell, hobnail, and Warthin-like) and hindered the statistical analysis in several subtypes, warranting further multicenter studies with a large sample size to better illustrate the difference in US findings.

In conclusion, US features of certain subtypes help suggest the differential diagnosis; however, definitive subtyping is not yet possible using US images alone. In the era of personalized medicine and active surveillance, acquaintance with the US characteristics of subtypes assumes greater importance for individualized treatment, and we believe our results may provide baseline information and stimulate future research in this field.

## Data Availability

The original contributions presented in the study are included in the article/supplementary material. Further inquiries can be directed to the corresponding author.
